# Multiple Negative Emotions During Learning With Digital Learning Environments – Evidence on Their Detrimental Effect on Learning From Two Methodological Approaches

**DOI:** 10.3389/fpsyg.2019.02678

**Published:** 2019-12-03

**Authors:** Franz Wortha, Roger Azevedo, Michelle Taub, Susanne Narciss

**Affiliations:** ^1^LEAD Graduate School and Research Network, University of Tübingen, Tübingen, Germany; ^2^Multimodal Interaction Lab, Leibniz-Institut für Wissensmedien, Tübingen, Germany; ^3^Department of Learning Sciences and Educational Research, University of Central Florida, Orlando, FL, United States; ^4^Psychology of Learning and Instruction, Faculty of Psychology, Dresden University of Technology, Dresden, Germany

**Keywords:** emotions, learning, digital learning environments, person-centered, variable-centered, emotion-regulation

## Abstract

Emotions are a core factor of learning. Studies have shown that multiple emotions are co-experienced during learning and have a significant impact on learning outcomes. The present study investigated the importance of multiple, co-occurring emotions during learning about human biology with MetaTutor, a hypermedia-based tutoring system. Person-centered as well as variable-centered approaches of cluster analyses were used to identify emotion clusters. The person-centered clustering analyses indicated three emotion profiles: a positive, negative and neutral profile. Students with a negative profile learned less than those with other profiles and also reported less usage of emotion regulation strategies. Emotion patterns identified through spectral co-clustering confirmed these results. Throughout the learning activity, emotions built a stable correlational structure of a positive, a negative, a neutral and a boredom emotion pattern. Positive emotion pattern scores before the learning activity and negative emotion pattern scores during the learning activity predicted learning, but not consistently. These results reveal the importance of negative emotions during learning with MetaTutor. Potential moderating factors and implications for the design and development of educational interventions that target emotions and emotion regulation with digital learning environments are discussed.

## Introduction

Learning is a complex multi-faceted process that requires students to deploy, monitor, and regulate their cognitive, metacognitive, affective and motivational processes based on the learning environment and the learning task and goal (Azevedo et al., 2018). Emotions play a central role in this context. They significantly impact and drive processes that are quintessential to learning, such as attention, perception, memory (Lewis et al., 2008; Tyng et al., 2017), and metacognition (Azevedo et al., 2017). Furthermore, a long tradition of research has shown that emotions are directly related to learning outcomes and academic achievement (Boekaerts and Pekrun, 2015). Even though initial investigations on emotions and learning has almost exclusively focused on the importance of anxiety in learning and test situations (Pekrun et al., 2002), research on emotions and learning has diverged into investigations of a broad variety of affective states and emotions in differing learning contexts (e.g., classroom settings, research with advanced learning technologies or informal learning settings; Azevedo et al., 2019). These studies have demonstrated that many different emotions are commonly experienced in learning settings (e.g., boredom, confusion, or frustration; D’Mello, 2013) and they have a significant impact on students’ performance (e.g., Pekrun et al., 2002; D’Mello et al., 2014). However, some important aspects of emotional experiences still have not been extensively researched in learning contexts. For example, most of the research in this context, particularly research during learning with digital learning environments, focused on the importance of single discrete emotions or sets of discrete emotions using variable-centered approaches. Research investigating emotions in other contexts, on the other hand, has revealed that approaches that consider multiple emotions simultaneously show great promise (e.g., Fortunato and Goldblatt, 2006; Vansteenkiste et al., 2009). Only a few studies have investigated the complexity of students’ (co-occurring) emotional experiences during learning using person-centered approaches (Ganotice et al., 2016; Jarrell et al., 2016, 2017; Robinson et al., 2017; Sinclair et al., 2018). These studies have found that groups of students who differ in their emotional experiences during learning in regard to multiple emotions (so called emotion profiles) also meaningfully differ in their learning outcomes and academic achievement. The goal of this study was to combine person- and variable-centered approaches to examining emotions during learning with a digital learning environment. We extended upon previous research by considering a broader range of emotion measures than previous studies (i.e., academic achievement emotions and learning-centered emotions), incorporating emotion regulation and temporal dynamics of emotions, and by substantiating person-centered analyses with a novel variable-centered approach.

### Emotions During Learning With Digital Learning Environments

Emotions are an essential component of learning activities across settings. Students’ emotional experiences when learning with technologies are diverse, have been investigated on the basis of several frameworks (D’Mello, 2013), and have been classified in various categories, including academic achievement emotions (Pekrun et al., 2002), epistemic or learning-centered emotions (D’Mello and Graesser, 2012; Pekrun et al., 2017b), and basic emotions (Ekman and Friesen, 1971; Ekman, 1992). Pekrun et al. (2002) and Pekrun (2006) academic achievement emotions approach distinguishes academic emotions differing in their valence (positive vs. negative) and the perceived level of control by the learner, including enjoyment (positive and high control), anxiety (negative and medium control), and hopelessness (negative and low control). Learning-centered emotions approaches (also referred to as cognitive affective states or epistemic emotions; D’Mello and Graesser, 2012; Muis et al., 2015; Pekrun et al., 2017b) focus on emotions that are directly related to knowledge-generating aspects of cognitive processes (e.g., overcoming impasses during learning), including boredom, confusion and frustration. According to Ekman (1992) six basic emotions can be distinguished across cultural contexts and reliably identified from facial expressions, including anger, happiness, and surprise. An extensive amount of research has shown that emotions significantly impact learning processes, outcomes, and academic achievement (Pekrun and Linnenbrink-Garcia, 2014). The majority of studies revealed that the way emotions impact learning and achievement is closely related to their valence. More specifically, positive emotions are positively, and negative emotions are negatively related to the learning process and learning outcomes (e.g., Pekrun et al., 2002, 2017a; Pekrun and Linnenbrink-Garcia, 2012). However, there is also evidence opposing this general pattern. For example, studies identified detrimental effects of positive emotions on the accuracy of metacognitive judgments creating an illusion of learning (Baumeister et al., 2015). Negative emotions on the other hand were positively associated with learning when they triggered deep processing of contents and were resolved by the students in a timely manner (see below, e.g., D’Mello and Graesser, 2014). This state of research indicates that, despite the overall tendency of beneficial effects of positive emotions and detrimental effects of negative emotions, further factors need to be considered to predict and explain the effects of emotions during learning.

A particular branch of research investigates (self-regulated) learning processes when learning with digital learning environments (Gegenfurtner et al., 2019), including hypermedia learning environments (e.g., Opfermann et al., 2013), intelligent tutoring systems (e.g., Azevedo et al., 2016; Harley et al., 2017), and game-based learning environments (e.g., Sabourin and Lester, 2014; Taub et al., 2018). These learning technologies have been designed and implemented to foster student learning about specific topics and have been shown to meaningfully enhance learning (Zheng, 2016). Digital learning environments include specific affordances that are directly linked to students’ emotions. For example, research has demonstrated that the design of digital learning environments (e.g., shapes and colors; Plass et al., 2014), their structure (e.g., complex, non-linear structure; Arguel et al., 2019), and scaffolds incorporated in such systems (e.g., prompts and feedback by pedagogical agents; Harley et al., 2017) can impact students’ emotions. More specifically, digital learning environments can elicit and alter emotional processes or assist the learner in regulating them and provide unique opportunities for research to investigate emotions in ways hardly achievable in other contexts. For instance, multi-channel trace data can be collected with digital learning environments to measure emotions with minimal interruptions to the learning process (e.g., through automated detection of facial expressions; D’Mello, 2017; Azevedo et al., 2019). The dynamics of affective states model is a prominent theoretical framework in this line of research that focuses on the dynamic unfolding of specific, learning-centered emotions (D’Mello and Graesser, 2012)^1^. More specifically, D’Mello and Graesser (2012) posited that confusion is elicited by impasses encountered during complex learning processes. This confusion can be beneficial to learning when it can be resolved, and the impasse can be overcome. Prolonged experiences of confusion on the other hand is theorized to lead to frustration and eventually boredom, which ultimately lead to disengagement and poor learning outcomes. Given that digital learning environments challenge students with learning tasks that require to develop a deep understanding of science concepts, or a solution for complex problems, such impasses are particularly likely to occur when learning with these systems. D’Mello and Graesser (2014) found a positive relation between (partially) resolved confusion and learning in a problem-solving task and a scientific reasoning task in an intelligent tutoring system. Another study by Taub et al. (2019) furthermore showed that the experience of frustration was linked to higher accuracy in the use of cognitive learning strategies (i.e., note-taking) with MetaTutor. However, they did not find a significant relation between emotions and learning gain.

Other studies on emotions and learning with digital learning environments (e.g., intelligent tutoring systems and game-based learning environments) on the other hand found detrimental effects of negative emotions. Initial studies on the relation between emotions and learning in AutoTutor identified significant detrimental effects of boredom for learning (Craig et al., 2004; Graesser et al., 2008). Across three studies using different digital learning environments, Baker et al. (2010) found further support for these findings by showing that boredom was the most persistent emotion (i.e., students were unlikely to transition from boredom to another emotion), and that boredom was the only emotion to be associated with maladaptive behaviors (i.e., gaming the system). Sabourin and Lester (2014) identified a positive relation between positive emotions and learning gains. Furthermore, they observed a negative association of confusion and boredom with learning gains in a game-based learning environment. A study by Grafsgaard et al. (2014) revealed that indicators of facially expressed frustration were negatively predictive of learning gain.

Taken together, these studies demonstrated the importance of learning-centered emotions during learning with digital learning environments (for a recent review see Arguel et al., 2019). However, they also demonstrated a profoundly controversial relation between (negative) emotions and learning. This clearly indicates further research is needed to disentangle the manifold relation between emotions, learning, and learning outcomes by identifying factors that explain these contradictory relations. One such factor that has been rarely considered in the aforementioned studies on emotions in digital learning environments is the co-occurrence of emotions. Even though studies have shown that the emotions outlined above have differential effects on learning depending on other affective states they are accompanied by or lead to (e.g., D’Mello and Graesser, 2012; Goetz et al., 2014; Riemer and Schrader, 2019), the co-occurrence of emotions and the breadth of emotional experiences has rarely been considered in this context.

## Person Centered Approaches to Emotions

Research on emotions during self-regulated learning has indicated that a variety of emotional states and processes impact learning and performance in meaningful ways. While these studies have greatly contributed to a comprehensive understanding of emotions in learning situations, especially when learning with digital learning environments, they have not fully considered the breadth of emotional experience of an individual. More specifically, the variable-centered approach used by these studies focuses on singular emotional states or a pre-selected set of emotions while controlling for the impact of other emotions. Emotion research on the other hand suggests that individuals can experience multiple emotions concurrently, and that these emotions affect each other reciprocally, which ultimately impacts thoughts and behaviors (e.g., Lazarus, 2006; Fernando et al., 2014). Person-centered approaches typically identify groups of students with similar emotional experiences in regard to multiple emotions at a certain point of time (often referred to as emotion profiles). These profiles are then compared to another and related to relevant outcome measures (e.g., learning and academic achievement). For example, multi-level investigations of affect in college students have revealed that spurs of negative emotions coupled with positive trait affectivity are associated with greater academic growth than positive or negative affect alone (Barker et al., 2016). Furthermore, the added value of this approach has been repetitively shown outside of educational contexts (e.g., Vansteenkiste et al., 2009; Fernando et al., 2014). In research in education settings, this approach is still quite rare. We identified five studies that used a person-centered analytical approach in different educational contexts (see Table 1 for a brief overview).

**TABLE 1:** Overview of person-centered studies on emotions during learning.

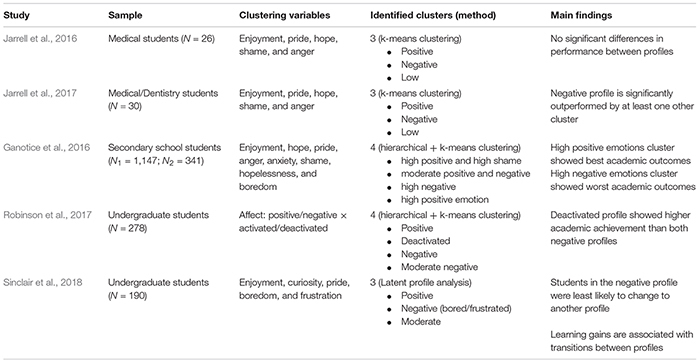

Jarrell et al. (2016, 2017) investigated emotions when learning with a computer-based learning environment using a person-centered approach in two studies. Five discrete emotional states (enjoyment, pride, hope, shame, and anger) measured with the Achievement Emotions Questionnaire (AEQ; Pekrun et al., 2002) were used to cluster students with similar emotional experiences. In both studies, a three-profile solution including a positive, negative, and low emotional experience profile, was identified. These profiles were subsequently related to learning outcomes. The first study (*N* = 26) revealed no significant differences in performance between profiles. In the follow-up study (*N* = 30) Jarrell et al. (2017) investigated differences in diagnostic performance efficiency between emotion profiles. They found that the negative emotion profile was outperformed by at least one other profile averaged across levels of difficulty (easy, medium, hard) and for easy and hard tasks, but not for tasks with medium difficulty.

Further investigations of emotions through a person-centered approach were conducted by Ganotice et al. (2016) in two secondary school samples. Similar to the studies outlined above, discrete emotional states (enjoyment, hope, pride, anger, anxiety, shame, hopelessness, boredom) measured through the AEQ (Pekrun et al., 2002) were used for clustering. In a domain general or a math-specific context, four emotion profiles were identified. These profiles included a high positive and high shame profile, a moderate positive and negative emotion profile, a high negative emotion profile, and a high positive emotion profile. These profiles were compared in regard to school engagement, motivation, and math performance. Results showed that profiles with high positive emotions were the most adaptive profiles while the high negative emotion profile was the least adaptive.

Robinson et al. (2017) investigated affective profiles in an undergraduate anatomy course. Other than previous person-centered studies, this research used two dimensions of affect (positive/negative × activated/deactivated, see Ben-Eliyahu and Linnenbrink-Garcia, 2013) as clustering variables. Through a two-step procedure, they identified four emotion profiles including a positive, a deactivated, a negative, and a moderate negative profile. Comparison in academic achievement revealed that the deactivated profile showed higher academic achievement than both negative profiles (negative and moderate negative) throughout three exams. Robinson et al. (2017) also found differences between the positive and the negative profile, but not throughout all exams. Lastly, they investigated the mediating role of (dis-) engagement and found that higher levels of performance for the positive and deactivated profile were mediated through lower levels of disengagement.

Lastly, Sinclair et al. (2018) investigated emotion profiles displayed in an undergraduate student sample that learned about the human circulatory system using MetaTutor (see 5.3 MetaTutor). They used five discrete emotion states (enjoyment, curiosity, pride, boredom, and frustration) measured at five time points before and during learning using latent profile analysis. Similar to the studies above, they found a positive, negative (bored/frustrated), and moderate emotion profile. Subsequently they investigated transitions between profiles and found that students from the negative profile were least likely to transition to another profile. Lastly, they found that learning gain predicted the transitions between profiles at specific, selected time points.

Taken together, these studies demonstrate that a person-centered approach can reveal emotion profiles across contexts, ranging from laboratory studies to research in schools and university. Furthermore, all studies have found that these profiles are significantly related to performance, academic achievement, and related constructs. Most of the previous studies have not incorporated learning-centered or epistemic emotions (e.g. boredom, confusion, and frustration; D’Mello and Graesser, 2012). On the other hand, previous research on emotions when learning with digital learning environments has found that these emotions significantly impact learning in varying ways. The finding that these emotions can have a positive or negative impact on learning is particularly interesting for person-centered research as the contradicting implications might be explained though co-occurring emotions (i.e., profiles that show similar levels of confusion or frustration, but varying levels of other emotions). The only study that investigated learning-centered emotions (Sinclair et al., 2018) on the other hand did not consider achievement emotions in their analysis, which makes comparisons across studies difficult. We aim to address this issue by including learning-centered emotions in addition to academic achievement emotions that were used in most of the person-centered studies outlined so far.

Furthermore, the aforementioned studies have investigated different constructs related to emotions and performance such as motivation (Ganotice et al., 2016) or engagement (Robinson et al., 2017) to substantiate their findings. None of the studies investigated the role of emotion regulation in this context. Emotion regulation is an essential component to emotional experiences in learning contexts and is a critical link between emotional experience and academic outcomes (Gross, 2015; Harley et al., 2019). It describes students’ efforts to influence which emotions they experience, when they experience these emotions and how they express them (Harley et al., 2019). Emotion regulation strategies are for example the cognitive reappraisal of emotional experiences or modification of the situation that elicited the emotion (Gross, 2015). Spann et al. (2019) found that emotion regulation significantly influenced the relation between emotions and learning in a game-based learning environment. More specifically, they found that cognitive reappraisal led to higher learning outcomes for highly confused, frustrated, and engaged students, but was not as effective for students with low levels of confusion, frustration and engagement. Incorporating emotion regulation could shed light on the development of emotions in relation to specific profiles. Adaptive profiles (such as described by Ganotice et al., 2016) are potentially defined by higher levels of emotion regulation to cope with high levels of negative emotions. To investigate this subject matter, temporal investigations of emotions related to emotion profiles similar to Sinclair’s approach (Sinclair et al., 2018) are necessary. This includes, investigating the self-reported use of emotion regulation strategies for the different emotion profiles, and exploring to what extent the intensity of emotional experiences fluctuates over time within profiles.

Lastly, the studies outlined above were limited to using person-centered approaches only. While the great value of this type of research has been shown, we argue that supplementing person-centered with other approaches can be essential to their understanding. More specifically, identifying if the distinguishing characteristics of profiles (e.g., varying levels of positive or negative emotion intensity) can be replicated through variable-centered approaches can provide additional insight on the origin of these profiles. Such approaches could differentiate if profiles are based on natural co-occurrence of emotions (e.g., high correlations between negative emotions) or specific combinations of individual emotional experiences (e.g., a profile with high levels of boredom and other negative emotions versus a profile with high levels of boredom and low levels of other negative emotions). Furthermore, replicating results using two different methodologies would reveal their level of robustness, which is particularly important in this context, because emotion profiles are identified through data driven approaches (guided by previous research).

## Current Study

The current study aims to address the issues outlined above by identifying emotion profiles of students who learned with MetaTutor and relate them to learning outcomes. To this end we decided to adapt the person-centered analytical procedure outlined by Vansteenkiste et al. (2009) and Robinson et al. (2017) for the identification of emotion profiles. Additionally, we demonstrate how a variable-centered approach can substantiate these results by relating patterns of emotions to emotion profiles and learning outcomes throughout different phases of learning (i.e., before the learning phase, at the start of the learning phase, and at the end of the learning phase, see section Emotion Items). More specifically, we aim to answer the following questions.

1.1 *Which emotion profiles can be identified during SRL with MetaTutor and how can they be described?* Given that the specific profiles are highly dependent on the number of clusters, no specific hypothesis can be formulated *a priori*. However, based on previous person-centered studies, we expect a negative and positive emotion profile (see Ganotice et al., 2016; Jarrell et al., 2016, 2017; Robinson et al., 2017; Sinclair et al., 2018). Additionally, further likely profiles can include a low-intensity or moderate intensity profile for all emotions.

1.2 *Are there significant differences in learning outcomes between the profiles?* Based on previous research, we expect the profile with the highest values of negative emotions to display the lowest learning gain (Ganotice et al., 2016; Jarrell et al., 2016, 2017; Robinson et al., 2017).

1.3 *Are there significant differences in self-reported use of habitual emotion regulation strategies between the profiles?* Based on research on emotion regulation, we expect profiles characterized by high negative emotion intensities to indicate lower levels of self-reported use of emotion regulation strategies (Harley et al., 2019).

2.1 *How can stable patterns of emotions can be identified throughout the different phases of the learning session and how can they be described?* Similar to our first research question, we expect a strong differentiation between negative and positive emotions in the different phases. Additionally, a strong differentiation between activating and deactivating emotions is expected (Ben-Eliyahu and Linnenbrink-Garcia, 2013). Furthermore, because neutral – per definition – refers to the absence of perceivable and detectable emotions, we hypothesize neutral to represent its own cluster (potentially paired with emotions that show low intensities overall). Lastly, based on the reoccurring finding that specific emotions are positively and/or negatively related to learning, we expect boredom, confusion or frustration to form separate cluster(s) from other negative emotions (e.g., D’Mello and Graesser, 2012).

2.2 *How are emotion profiles related to the phase-specific patterns of emotions?* We expect emotion profiles to significantly differ in regard to emotion clusters that are defined by valence as all previous studies included profiles that were defined by positive and negative emotions (Ganotice et al., 2016; Jarrell et al., 2016, 2017; Robinson et al., 2017). In an exploratory step we will investigate if these differences are stable over time or if they arise throughout specific parts of the learning session.

2.3 *How can the phase-specific patterns of emotions predict learning outcomes in the respective phases of the learning activity?* Based on previous research, we expect negative emotions to be most predictive of learning. However, the direction of this interaction will be explored, as previous research has shown controversial results in this regard.

## Materials and Methods

### Participants

One hundred ninety-four (*N* = 194) undergraduate students (aged between 18 and 41, *M* = 20.46 years, *SD* = 2.96 years; 53% female) from three large public North American universities participated in a 2-day laboratory study. They were randomly assigned either to the *prompt and feedback* (P + F) or *control* (C) condition (see section MetaTutor), and monetarily compensated for their time ($10 per hour, up to $40). For the present study, only participants that filled out a sufficient number of emotion questionnaires (see section Emotion Items) were included in analyses, resulting in a sample size of one hundred seventy-six (*N* = 176) students.

### Procedure

The experiment was conducted over 2 days. On the first day, participants signed a consent form, filled in demographics questions, and completed several self-report measures (e.g., the Achievement Emotions Questionnaire – Pekrun et al., 2002 and the Emotion Regulation Questionnaire – Gross and John, 2003). Lastly, after responding to the questionnaires, participants took a 30-item pretest about the human circulatory system.

On the second day of the experiment, students were first introduced to the learning task and learning environment. They were instructed to set two learning sub goals before the beginning of the learning phase. During the learning phase, participants had to engage in self-regulated learning by reading texts, inspecting corresponding diagrams, and completing quizzes. Moreover, regardless of the experimental condition (see section MetaTutor) students were free to indicate their use of certain cognitive (e.g., note taking) or metacognitive learning strategies and activities using the SRL palette implemented in MetaTutor’s interface (see section MetaTutor). Additionally, quizzes and self-report measures (i.e., the emotion and values [EV] questionnaire; Azevedo et al., 2013) were administered based on specific rules implemented by the system (e.g., the EV was conducted on a time-based threshold – roughly every 14 min during the learning session with MetaTutor).

After the 60-min learning phase, students were directed to the post test (i.e., 30-item test about the circulatory system) and had to complete a last set of self-reports (e.g., an EV directly before the posttest) before they were debriefed by the research assistant.

During the experiment, multiple channels of multimodal data, including eye tracking, galvanic skin response, and automated analysis of facial expressions were collected. However, these process measures were not analyzed in the present study.

### MetaTutor

**FIGURE 1:**
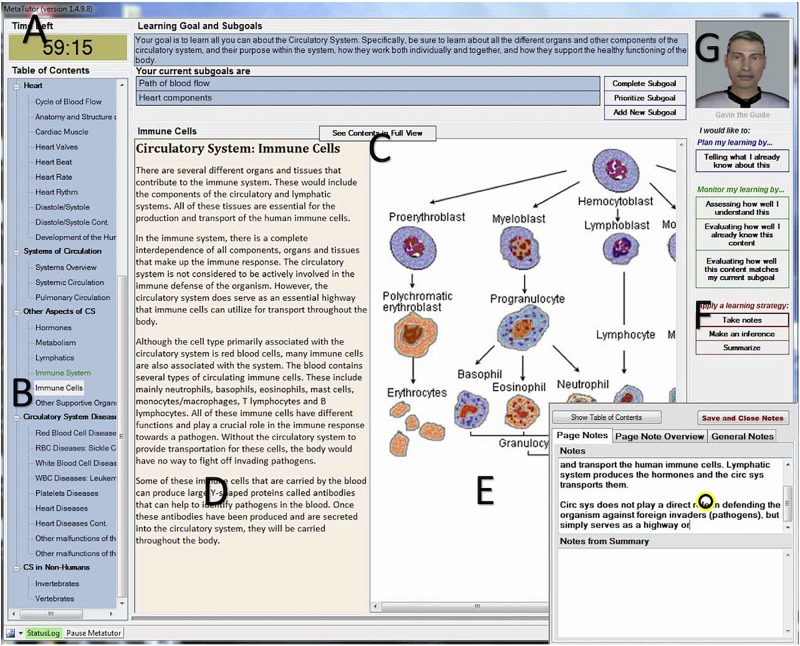
Screenshot of the MetaTutor interface. **(A)** Timer, **(B)** Table of contents, **(C)** Progress bar, **(D)** Content text, **(E)** Content image, **(F)** SRL palette, and **(G)** The pedagogical agent.

MetaTutor is a hypermedia-based tutoring system that fosters self-regulated learning processes while learning about the human circulatory system (Azevedo et al., 2018). The system was designed using a set of production rules, which fire based on how students monitor and control their understanding of the text and relevancy of the current page to the sub-goal they are working on. In addition to the processes being prompted by the pedagogical agents based on the production rules, participants were able to engage in any process of their choice. The MetaTutor learning environment was strategically designed to foster the use of cognitive learning strategies and metacognitive monitoring processes (see Figure 1). For example, a timer (A) and sub goal progress bar (C) allow students to monitor their progress toward achieving their sub goals and overall learning goal. The table of contents (B) provides students all the content page titles so they can select the appropriate pages to read for achieving their sub goals. There are seven pre-set sub goals in the environment (path of blood flow, heartbeat, heart components, blood vessels, blood components, purposes of the circulatory system, and malfunctions of the circulatory system). Prior to the 60-min learning session, students are progressed through a sub goal setting phase where they are guided to set two of those sub goals. The content text (D) and diagram (E) facilitate knowledge acquisition and foster coordinating information between the text and diagram. The SRL palette (F) provides students the opportunity to select cognitive learning strategies (i.e., prior knowledge activation, take notes, summarize, make an inference) and metacognitive monitoring processes (judgment of learning, feeling of knowing, content evaluation) they want to use during learning about the human circulatory system.

There are four pedagogical agents with one present at a time (G), where each agent focuses on a specific component of SRL. Gavin (shown in Figure 1) guides students through the learning environment and administers self-report questionnaires. Pam fosters planning by helping students set sub goals and activate their prior knowledge. Sam focuses on strategy use. Mary emphasizes monitoring processes. The amount of assistance provided by the pedagogical agents depends on the experimental condition students are assigned to. In the P + F condition, the agents prompt students to engage in SRL processes (using time- and event-based production rules). They also provide feedback on how they performed. For example, Sam will prompt students to make a summary, and once they have done so, he will tell them it is too long, too short, acceptable, etc. In the C condition, students are not prompted by the agents, nor are they given any feedback on their performance. In this condition, students can still initiate the use of cognitive and metacognitive processes, however, they are still not given any feedback, whereas in the P + F condition, students can also self-initiate the use of these processes, and will be given feedback on their performance.

### Measures

#### Emotion Items

Students’ emotional experiences at the start, during, and at the end of the learning session were measured using the Emotion-Values Questionnaire (EV; Azevedo et al., 2013). The EV covers 15 emotional states as well as two questions asking about the perceived value and the students’ ability to perform well on the current task on a five-point Likert-scale (ranging from 1 – “Strongly Disagree” to 5 – “Strongly Agree”). Additionally, two forced choice items asked the participants to select the emotion that best describes how they currently feel out of 15 (all emotional states from the EV) and 7 options (basic emotions), respectively. The emotional states included in the EV were based on extensive research on achievement emotions in academic settings (Pekrun et al., 2002; Pekrun, 2006), as well as on research on learning-centered emotions/epistemic emotions (e.g., D’Mello and Graesser, 2012; Muis et al., 2015; Pekrun et al., 2017b). The questionnaire covers the following emotions (in order of administration): enjoyment, hope, pride, frustration, anxiety, shame, hopelessness, boredom, surprise, contempt, confusion, curiosity, sadness, eureka, and neutral. A definition and an example were provided for each emotional state during each administration.

The EV was administered at fixed points of time before and after the learning phase, and time-based during the learning phase. More specifically, the questionnaire was administered directly before and after participants set their learning sub goals, and before the actual learning phase. During the learning activity the questionnaire was administered every 14 min. Lastly, the final EV was administered when the learning phase was finished, directly before the post test. The number of EVs completed varied between participants because the administration during the learning phase was postponed when key learning activities took place. In particular, the questionnaire did not interrupt any of the user- or agent-initiated learning strategies that required completing quizzes or filling out questionnaires. For example, if a student initiated the sequence of finishing the current learning sub goal, they had to fill out a 10-item multiple-choice quiz on the current topic and received feedback depending on the experimental condition (see section MetaTutor). If an EV should have been administered during that sequence, it was postponed until the end of the sequence, potentially delaying it by several minutes. This resulted in a range of four to eight EVs completed between participants. To allow for comparisons of participants, we decided to limit the EVs analyzed in the present study to six points of time relative to the start and the end of the learning session. Therefore, only participants that completed at least six EVs were considered for analyses, resulting in a final sample size of one-hundred-seventy-six students (*N* = 176). The following EVs were selected: (1) the first two EVs that were completed at the beginning and end of the sub goal setting phase, (2) the third and fourth EV, which took place in the first half of the learning phase, and (3) the last two EVs, which was the last questionnaire presented during the learning phase, and the final EV immediately prior to the post test. Due to missing data, “Eureka” was excluded from analyses in the current study, yielding 14 discrete emotions considered for analyses.

#### Pre and Post Tests

Prior knowledge and learning outcomes were measured using two 30-item multiple choice tests covering conceptual knowledge of the human circulatory system. The measures were developed by a domain expert in the subject matter. Each question had four potential answers and one correct solution. The order of two equivalent versions of the tests was randomized and counterbalanced across experimental conditions. Percent correct for both measures were computed for analyses.

#### Emotion Regulation Questionnaire

Students’ self-reported habitual use of emotion regulation strategies was measured using the emotion regulation questionnaire (ERQ; Gross and John, 2003). The 10-item questionnaire features two sub-scales asking about the use of emotion regulation strategies using a seven-point Likert scale (ranging from 1 – strongly disagree to 7 – strongly agree). More specially, mean values for the sub-scales expressive suppression (4 items, α = 0.78; e.g., “I keep my emotions to myself.”) and cognitive reappraisal (6 items, α = 0.84; e.g., “I control my emotions by *changing the way I think* about the situation I’m in.”) were calculated for analyses.

### Statistical Analyses

Statistical analyses in the present study were conducted using R (R Core Team, 2019), Python (Van Rossum and Drake, 2011), and SPSS (SPSS, 2012). Before the initial analyses, we investigated if the mean scores for each emotion computed over the six administrations of the EV for clustering contained significant outliers using Grubbs (1969) approach (implemented through the ‘grubbs.test’ function of the outlier package for R; Komsta, 2011). In total, 12 univariate outliers were replaced by the closest non-outlier value (three for shame, one for hopelessness, two for surprise, two for confusion, and five for surprise). Furthermore, investigations of the skewness and kurtosis (values < 2; George and Mallery, 1999) revealed that all of the variables used for analyses (i.e., mean emotion scores, emotion cluster scores and learning measures) were within acceptable ranges of normal distribution.

The person-centered methodological approach for the identification of emotion profiles was based on previous studies investigating affective, emotional, or motivational profiles (Vansteenkiste et al., 2009; Robinson et al., 2017). More specifically, we first used the ‘hclust’ function of R’s stats package to compute a range of profile solutions using Ward’s method and extracted the cluster centroids for each profile. We used agglomeration coefficients obtained through the SPSS classification function (SPSS, 2012), minimum number of profile size (Fernando et al., 2014), and cluster fit indices from ‘Nbclust’ (Charrad et al., 2014) to identify the eligible range of clusters. Subsequently, k-means clustering analysis with these centroids as starting points was conducted (‘kmeans’ function of the ‘stats’ library) to obtain the most distinctive set of profiles. As a last step in the cluster identification we used the cross-validation procedure outlined by Breckenridge (2000) to assess the stability of the solution (using self-implemented function based on the ‘knn’ function of the ‘class’ library; Venables and Ripley, 2002). Together with investigations of explained variance in the clustering variables and redundancy of the clusters, this criterion was used to determine the final cluster solution. Clustering methodology was chosen because the suitability of clustering over other methodological approaches in this context has been repeatedly showcased by previous research (e.g., Robinson et al., 2017).

Subsequently we used a latent growth linear mixed effect model to investigate differences in learning outcomes between emotion profiles. Models were fit using ‘lmer’ from the ‘lme4’ library (Bates et al., 2014). Summary statistics were extracted via the ‘analyze’ function of ‘psycho’ (Makowski, 2018) and *post hoc* comparisons were conducted using ‘glht’ from ‘multicomp’ (Hothorn et al., 2008). Additionally, this analysis was repeated for all profile solutions (including the initial solutions from hierarchical clustering) to assess if the findings were stable throughout different profile configurations.

Then spectral co-clustering – a machine learning clustering approach – implemented through the ‘SpectralCoclustering’ function of the Python library ‘scikit-learn’ was used to substantiate the relation between emotions and learning identified through the profiling approach (Pedregosa et al., 2011). Specifically, we grouped emotions into clusters based on their correlation across all measurement points and separately for each time point. The emotion cluster solution was selected based on its stability over all administrations of the EV and alignment to previous research. Then, principal component analysis (‘PCA’ function of ‘scikit-learn’) with one main component was used to obtain participants’ scores for each emotion cluster at each measurement point. Additionally, the internal consistency of emotion clusters was assessed though Cronbach’s Alpha (‘alpha’ of R’s ‘psych’ package; Revelle, 2017). The obtained scores were then used in multiple regressions for each time point separately to assess how the emotion clusters are related to learning. Regression weights were calculated using the ‘lm.beta’ function R’s ‘lm.beta’ package (Behrendt, 2014).^2^

### Preliminary Analyses

To control for the potential effect of the experimental manipulation of the present study (i.e., the control and prompt + feedback conditions) on the results described in the following sections, all variables included in the analyses were compared between the experimental conditions using multivariate analyses of variance (MANOVAs). Results showed no systematic differences in pre- and posttest scores, emotion scores, or emotion cluster scores between the conditions (all *p* > 0.05; except negative emotions cluster scores for EV 1: *p* < 0.05). Additionally, we conducted chi-square tests for each profile solution to test if the experimental conditions were equally represented in each emotion profile. Results revealed no significant differences in the distribution of experimental conditions for any of the emotion profiles identified.

### Person-Centered Approach: Emotion Profiles

#### Identifying Emotion Profiles

To identify emotion profiles, students with similar self-reported emotional experiences were grouped using a two-step clustering approach. More specifically, first, hierarchical clustering (Ward’s method) was used on the squared Euclidian distance matrix for the mean values of each emotion for each participant throughout all six time points (see above). Each participant started as their own cluster in the hierarchical clustering analyses. Then the closest participants were merged into a cluster. This step was repeated until all participants were merged into a single cluster, resulting in a range of cluster solutions between the number of participants (i.e., each participant as their own cluster) and a singular cluster. To identify the profile solutions eligible for subsequent analyses, we used three criteria: (1) the scree-plot of agglomeration coefficients to identify the point where the addition of clusters did not substantially decrease the agglomeration coefficient, (2) a sufficient profile size for statistical analyses (*n* > 10; Fernando et al., 2014), and (3) multiple cluster fit indices (Charrad et al., 2014). Agglomeration coefficient indicated that merging a three-cluster solution into two clusters was not practical (Δ_*coefficient*_ = 233.93). A second drop in agglomeration coefficients was identified for the addition of a sixth cluster, but was less substantial (Δ_*coefficient*_ = 73.379). While this procedure favored solutions with more than six profiles, the second criterion limited the number of profiles to a maximum of seven, as all further profile solutions included profile(s) with less than ten participants. Lastly, we compared the solutions that were sufficient for both criteria in regard to 26 fit indices (see Charrad et al., 2014 for a complete list of the indices) and found equal support for the three to five profile solutions and little to no support for the six and seven profile solutions. Accordingly, the three-, four-, and five-profile solutions were selected for further analyses.^3^ Preliminary analyses on the structure of the clusters revealed a noteworthy feature. A single emotion profile with higher negative emotion intensities than other profiles (*n* = 29) was a stable component of all solutions outlined above.

As a second step in the identification of emotion profiles we used k-means clustering, a non-hierarchical clustering procedure, in order to increase similarity within clusters and differences between clusters. More specifically, for the previously selected three- to five-cluster solutions, we first extracted the cluster centroids. These values were then used as starting points of the k-means clustering instead of starting with randomized seeds. In this procedure the number of clusters is defined *a priori*. Then a starting seed was used as the initial centroid of a cluster and participants that were in proximity to that centroid (measured through a distance threshold) were assigned to that cluster. This procedure was repeated for each starting seed until all participants were assigned to a cluster (Fortunato and Goldblatt, 2006). K-means clustering was chosen because this procedure simultaneously maximizes between cluster distances (i.e., increased differences between emotion profiles) and minimizes within-cluster variance (i.e., increased similarity within profiles; Eshghi et al., 2011). After obtaining the respective cluster solution, we then assessed the rate of agreement between the hierarchical and k-means approaches. Both clustering methods showed sufficient rate agreement (*K*_3_ = 0.76; *K*_4_ = 0.78; *K*_5_ = 0.78). This indicated that the k-means clustering altered the initial profiles obtained through the hierarchical clustering but maintained the overall structure and demonstrates the robustness of the identified profiles. To test if the aggregation of self-reported emotion intensities had a significant impact on the obtained emotion profiles, we re-ran all previous steps using all six measurement points for the fourteen emotions as clustering variables. Comparison of the profiles identified by clustering means and the profiles identified by clustering all measurement points demonstrated high to very high agreement (*K*_3_ = 0.85; *K*_4_ = 0.91; *K*_5_ = 0.88). This indicated that our data supports the use of mean values as clustering variables and further underlined the robustness of the clustering procedure.

To select the emotion profiles for subsequent analyses we first compared the explained variance in mean emotion intensities between the solutions with different numbers of emotion profiles. The three-profile solution explained moderate levels of variance for all mean emotion intensities, except neutral, surprise, anxiety and contempt (see Table 2). The four-profile solution explained more variance for most of the emotions, but also showed lower levels of explained variance for specific emotions (i.e., contempt and confusion). This pattern also applied to the comparison of the four- and five-profile solutions. However, while the four-profile solution added a profile that was primarily defined by boredom in addition to the neutral, positive, and negative emotion profiles of the three-profile solution, the five-profile solution only added a profile that was largely redundant to the positive emotion profile (with higher levels of curiosity, surprise and anxiety). Based on the largely redundant nature of this profile (a criteria used by Fernando et al., 2014), we decided not to consider this solution.

**TABLE 2:** Explained variance by profile-solution.

**Emotion**	**Profile solution**		
	**3**	**4**	**5**
Enjoyment	0.47	0.63	0.62
Hope	0.46	0.55	0.54
Pride	0.34	0.39	0.40
Frustration	0.31	0.38	0.41
Anxiety	0.20	0.22	0.42
Shame	0.55	0.60	0.65
Hopelessness	0.64	0.67	0.68
Boredom	0.44	0.60	0.62
Surprise	0.14	0.23	0.39
Contempt	0.22	0.13	0.12
Confusion	0.40	0.34	0.43
Curiosity	0.36	0.49	0.46
Sadness	0.39	0.44	0.45
Neutral	0.10	0.15	0.23
Average	0.36	0.42	0.46

**FIGURE 2:**
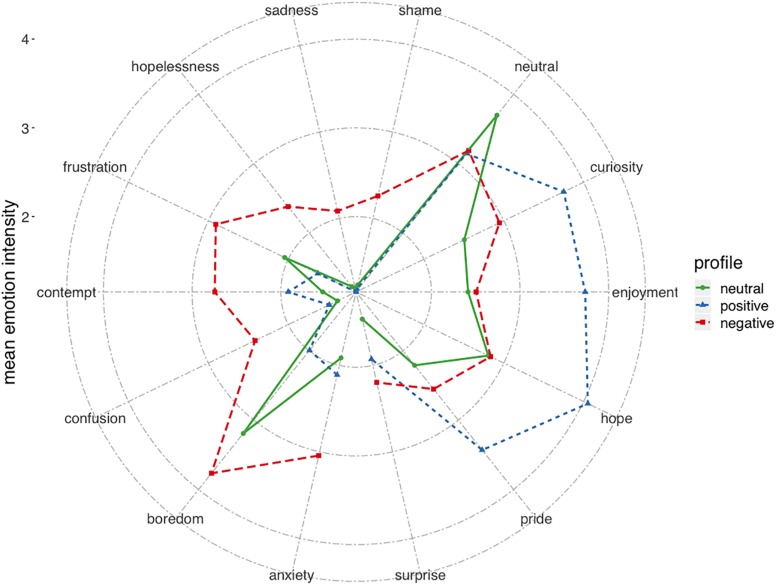
Comparison of mean emotion intensities between profiles.

As the final step for selecting the most suitable cluster solution, we cross validated the three- and four-profile solutions following the procedure outlined by Breckenridge (2000). More specifically, we split our sample randomly into two equally large sub samples. Then, the two-step clustering procedure outlined above was separately applied to each of the sub samples. The two sub samples were subsequently compared with a k-nearest-neighbors approach. More specifically, each participant of a sub sample was assigned to a new cluster value based on their most similar counterparts in the other sub sample (their nearest neighbors). To assess the robustness, Kohen’s Kappa (as a measure for agreement) was calculated based on the initial (obtained through the two-step approach) and new cluster assignment (obtained through the nearest neighbors procedure) in both samples. To increase the robustness of the cross-validation, we repeated this procedure twenty times and averaged Kappa values across all iterations (i.e., 20-fold cross validation). Results indicated that the three-profile solution (*K* = 0.65) showed sufficient stability (i.e., *K* > 0.60; Breckenridge, 2000; Asendorpf et al., 2001), but the four-profile solution did not (*K* = 0.56). Therefore, the three-profile solution was selected as the final profile solution (see Figure 2 for a comparison of mean emotion intensities between the three profiles). Means and standard deviations for mean emotion intensities, and pre and post test scores of the three-profile solution are displayed in Table 3. The three profiles can be described by their most distinct features as follows^4^. The first profile (*n* = 75) displayed low to moderate levels for all emotions except boredom and neutral, which were at moderate levels. The neutral score was higher than for the other profiles. Accordingly, we refer to this profile as *neutral*. The second profile (*n* = 62) showed moderate to high levels for most of the positive emotions (joy, hope, pride, curiosity) and low levels of negative emotions (frustration, shame, hopeless, boredom, contempt, confusion, and sadness). The positive emotion intensities were higher in this profile compared to those of the other profiles. Thus, we labeled this profile as the *positive* emotion profile. The final profile (*n* = 39) was characterized by medium levels for all emotions. When compared to the other profiles, the most distinct feature of this group was their increased levels of negative emotion intensities for all negative emotions. Therefore, we referred to this group as the *negative* emotion profile. A multivariate analyses of variance (MANOVA) revealed that the emotion profiles significantly differed in regard to their mean emotion intensities [Wilks’s λ (28, 320) = 0.100, *p* < 0.001, η^2^ = 0.68].

**TABLE 3:** Means and standard deviations for emotion items, emotion regulation, and learning measures by profile solutions.

**Profile solution**	**3**			**4**				**5**				
			
**Profile**	**1**	**2**	**3**	**1**	**2**	**3**	**4**	**1**	**2**	**3**	**4**	**5**

***n***	**75**	**62**	**39**	**27**	**67**	**50**	**32**	**27**	**60**	**27**	**32**	**30**

	***M (SD)***	***M (SD)***	***M (SD)***	***M (SD)***	***M (SD)***	***M (SD)***	***M (SD)***	***M (SD)***	***M (SD)***	***M (SD)***	***M (SD)***	***M (SD)***
Enjoyment	2.41 (0.67)	3.73 (0.62)	2.5 (0.66)	1.66 (0.42)	2.84 (0.53)	3.84 (0.59)	2.6 (0.59)	1.66 (0.42)	2.8 (0.51)	3.55 (0.63)	2.6 (0.59)	3.94 (0.62)
Hope	2.8 (0.71)	4.05 (0.55)	2.83 (0.64)	2.29 (0.66)	3.09 (0.61)	4.19 (0.51)	2.92 (0.58)	2.29 (0.66)	3.10 (0.6)	3.69 (0.63)	2.92 (0.58)	4.37 (0.49)
Pride	2.21 (0.77)	3.43 (0.78)	2.55 (0.65)	1.78 (0.55)	2.50 (0.74)	3.53 (0.81)	2.67 (0.63)	1.78 (0.55)	2.5 (0.71)	3.03 (0.96)	2.67 (0.63)	3.75 (0.67)
Frustration	2.04 (0.84)	1.63 (0.56)	2.91 (0.61)	2.72 (0.87)	1.76 (0.68)	1.67 (0.56)	2.92 (0.57)	2.72 (0.87)	1.77 (0.68)	1.93 (0.56)	2.92 (0.57)	1.43 (0.47)
Anxiety	1.91 (0.81)	2.11 (1.00)	3.04 (0.76)	2.12 (0.81)	1.88 (0.81)	2.16 (1.01)	3.19 (0.75)	2.12 (0.81)	1.76 (0.65)	3.00 (0.84)	3.19 (0.75)	1.57 (0.74)
Shame	1.23 (0.34)	1.19 (0.3)	2.26 (0.56)	1.29 (0.34)	1.22 (0.35)	1.2 (0.32)	2.41 (0.47)	1.29 (0.34)	1.19 (0.28)	1.48 (0.45)	2.41 (0.47)	1.02 (0.05)
Hopelessness	1.23 (0.33)	1.15 (0.26)	2.38 (0.55)	1.31 (0.38)	1.24 (0.34)	1.14 (0.25)	2.52 (0.47)	1.31 (0.38)	1.23 (0.34)	1.30 (0.34)	2.52 (0.47)	1.04 (0.09)
Boredom	3.19 (0.93)	1.99 (0.68)	3.76 (0.67)	4.24 (0.56)	2.73 (0.73)	1.91 (0.67)	3.66 (0.63)	4.24 (0.56)	2.83 (0.68)	1.88 (0.63)	3.66 (0.63)	1.93 (0.68)
Surprise	1.46 (0.48)	1.93 (0.88)	2.2 (0.68)	1.23 (0.33)	1.57 (0.49)	2.04 (0.91)	2.33 (0.65)	1.23 (0.33)	1.56 (0.49)	2.56 (0.8)	2.33 (0.65)	1.47 (0.59)
Contempt	1.53 (0.74)	1.92 (1.05)	2.74 (0.66)	2.01 (0.93)	1.60 (0.87)	1.88 (1.08)	2.65 (0.55)	2.01 (0.93)	1.63 (0.9)	1.88 (0.89)	2.65 (0.55)	1.75 (1.16)
Confusion	1.38 (0.43)	1.49 (0.53)	2.41 (0.56)	1.55 (0.65)	1.40 (0.43)	1.51 (0.55)	2.45 (0.55)	1.55 (0.65)	1.40 (0.42)	1.86 (0.54)	2.45 (0.55)	1.18 (0.3)
Curiosity	2.50 (0.66)	3.75 (0.81)	2.94 (0.76)	1.93 (0.59)	2.82 (0.62)	3.89 (0.74)	3.10 (0.64)	1.93 (0.59)	2.82 (0.59)	4.02 (0.58)	3.10 (0.64)	3.54 (0.95)
Sadness	1.19 (0.37)	1.17 (0.32)	2.09 (0.61)	1.28 (0.42)	1.16 (0.34)	1.19 (0.35)	2.23 (0.55)	1.28 (0.42)	1.16 (0.34)	1.29 (0.43)	2.23 (0.55)	1.10 (0.22)
Neutral	3.70 (0.71)	3.15 (0.83)	3.19 (0.75)	3.30 (0.86)	3.79 (0.61)	3.03 (0.84)	3.20 (0.7)	3.30 (0.86)	3.90 (0.53)	2.79 (0.77)	3.20 (0.7)	3.20 (0.8)
Reappraisal	4.95 (1.09)	5.30 (1.07)	4.62 (1.27)	5.03 (1.26)	5.00 (0.92)	5.36 (1.15)	4.41 (1.30)	5.03 (1.26)	5.03 (0.88)	5.16 (1.22)	4.41 (1.30)	5.38 (1.13)
Suppression	3.97 (0.97)	3.97 (1.13)	3.94 (1.17)	3.78 (0.98)	3.97 (1.04)	4.03 (1.11)	4.01 (1.16)	3.78 (0.98)	4.03 (1.04)	4.09 (1.05)	4.01 (1.16)	3.83 (1.15)
Pre ratio	0.59 (0.13)	0.58 (0.14)	0.55 (0.17)	0.53 (0.11)	0.60 (0.13)	0.59 (0.13)	0.55 (0.19)	0.53 (0.11)	0.60 (0.13)	0.59 (0.12)	0.55 (0.19)	0.59 (0.14)
Post ratio	0.71 (0.12)	0.70 (0.13)	0.63 (0.16)	0.67 (0.10)	0.72 (0.13)	0.7 (0.13)	0.62 (0.17)	0.67 (0.10)	0.72 (0.13)	0.71 (0.11)	0.62 (0.17)	0.70 (0.14)

*Reappraisal, cognitive reappraisal subscale of the emotion regulation questionnaire; suppression, expressive suppression subscale of the emotion regulation questionnaire.*

#### Linking Emotion Profiles and Learning Outcomes

**FIGURE 3:**
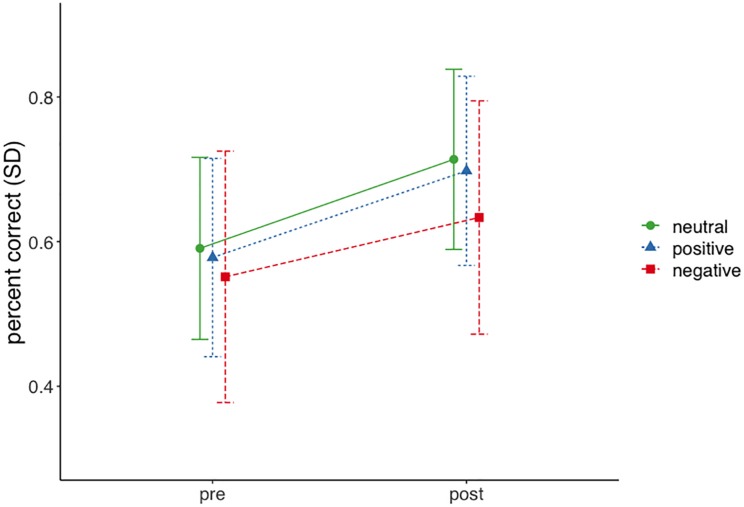
Pre and post test scores by emotion profile.

Differences in learning outcomes between profiles were analyzed using a latent growth linear mixed effect model. More specifically, we predicted learning outcomes with time (pre and post test) and profile membership as fixed factors and included a random intercept^5^ based on previous studies that showed the importance of individual differences in prior knowledge when learning with MetaTutor (Taub et al., 2014). The model explained significant proportion of variance in learning outcomes (*R*^2^ = 68.03%; fixed effects: *R*^2^ = 16.23%) and showed that learning outcomes significantly improved over time for all profiles [β = 0.75, *SE* = 0.06, *t*(175) = 12.36, *p* < 0.001, *VIF* = 1.00] and that membership in the negative profile was associated with significantly lower learning outcomes [β = −0.40, *SE* = 0.16; *t*(173) = −2.43, *p* < 0.05, *VIF* = 1.18; see Figure 3]^6^. *Post hoc* test using Tukey’s HSD (honestly significant difference) showed that significant differences in learning outcomes were only found between the negative and neutral profile (*z* = −2.432; *p* < 0.05).

#### Linking Emotion Profiles and Emotion Regulation

Two separate ANOVAs comparing expressive suppression and cognitive reappraisal between the profiles were conducted to test if profiles differed in their self-reported habitual use of emotion regulation strategies. Results showed that there were no significant differences in expressive suppression [*F*(2,163) = 0.013; *p* = 0.99], but significant differences in cognitive reappraisal between profiles [*F*(2,163) = 4.185; *p* < 0.05]. *Post hoc* comparisons using Bonferroni correction revealed that students with a negative emotion profile had significantly lower cognitive reappraisal scores (*M* = 4.62, *SD* = 1.27) then those with a positive emotion profile (*M* = 5.30, *SD* = 1.07; *p* < 0.05).^7^

### Variable-Centered Approach: Patterns of Co-occurring Emotions

#### Identifying Patterns of Co-occurring Emotions

To identify patterns of co-occurring emotions, correlation matrices for the 14 emotions investigated in this study were computed separately for each point of time (see procedure) and aggregated over all points of time. Then, spectral co-clustering, a clustering technique that groups data by rows and columns simultaneously (e.g., Kluger et al., 2003), was applied to these matrices to obtain the variable-centered patterns of related emotions for each point of time and aggregated over all EV administrations. This procedure was carried out for cluster solutions ranging from three to six clusters. A four-cluster solution was the only one that displayed great stability over all time points and aggregated over all measures (the only exception is that contempt moved to the boredom cluster during the last measurement). This solution included a positive and a negative emotions pattern, as well as neutral and boredom as singular-emotion clusters (see Table 4). Cronbach’s Alpha was calculated for the negative and positive emotions pattern separately for each time point to test if the identified cluster represented an internally consistent linear structure sufficiently well. Results showed that both the negative pattern (alpha ranging from 0.74 to 0.81) and the positive pattern (alpha ranging from 0.72 to 0.85) met this criterion.^8^ We obtained participants’ individual scores for each pattern and the maintained variance of each pattern through principal component analyses with one component. The maintained variance from the original Likert-scale items for each non-singular emotion pattern was sufficient in this solution (35.45% for the negative emotions pattern for EV2 and 68.40% for the positive emotions pattern for EV2, see Table 4). Loadings for all emotions were positive for each pattern (i.e., increases in emotion intensity was associated with an increase in pattern score).

**TABLE 4:** Maintained variance and loadings for emotion patterns.

**Pattern**	**Variable**	**Time point**						
		**Overall**	**EV T1**	**EV T2**	**EV T3**	**EV T4**	**EV T5**	**EV T6**
Negative	σ^2^	0.40	0.38	0.35	0.40	0.43	0.42	0.50
	Frustration	0.85	0.52	0.56	0.89	1.02	0.94	0.87
	Anxiety	0.83	0.95	0.74	0.82	0.86	0.84	0.95
	Shame	0.60	0.50	0.59	0.61	0.68	0.50	0.67
	Hopelessness	0.63	0.44	0.40	0.71	0.72	0.71	0.70
	Surprise	0.43	0.45	0.52	0.53	0.32	0.36	0.45
	Confusion	0.64	0.39	0.47	0.68	0.67	0.70	0.80
	Sadness	0.49	0.36	0.49	0.49	0.52	0.54	0.50
	Contempt^∗^	0.52	0.58	0.54	0.65	0.49	0.65	
Positive	σ^2^	0.65	0.55	0.68	0.65	0.66	0.63	0.66
	Enjoyment	0.94	0.73	0.85	0.94	0.92	1.06	1.02
	Hope	0.97	0.78	0.90	0.92	0.95	1.02	1.02
	Pride	0.86	0.77	0.89	0.89	0.88	0.87	0.98
	Curiosity	0.99	0.57	0.88	0.95	0.94	0.87	0.89
Neutral	σ^2^	1.00	1.00	1.00	1.00	1.00	1.00	1.00
	Neutral	1.16	1.08	1.15	1.06	1.12	1.15	1.15
Boredom	σ^2^	1.00	1.00	1.00	1.00	1.00	1.00	1.00
	Boredom	1.34	1.18	1.22	1.36	1.41	1.39	1.27
	Contempt^∗^							0.61

*σ^2^, maintained variance. EV, emotion values questionnaire. Absolute loading values were used if all loadings on the same main component were negative. ^∗^Contempt at EV T6 is the only deviation from the stable structure of emotions. It was associated with the boredom pattern instead of the negative pattern.*

#### Exploring Differences in Variable-Centered Emotion Patterns Scores Between Emotion Profiles

**FIGURE 4:**
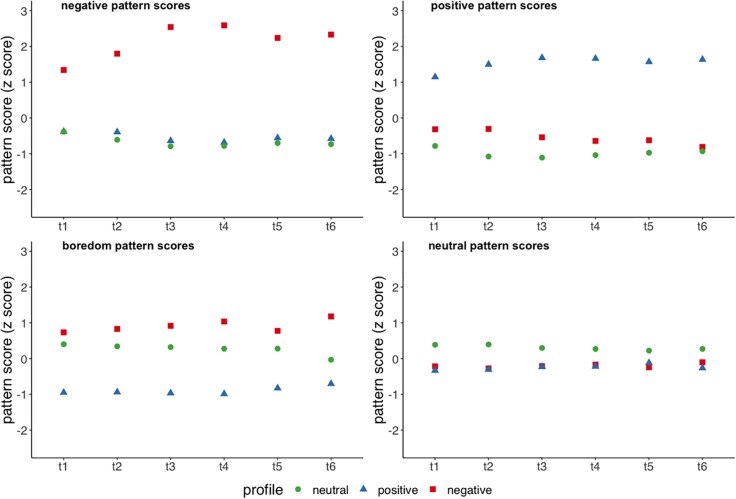
Emotion pattern scores by emotion profile over the six measurement points.

Differences in emotion variable-centered cluster scores between profiles over time were analyzed using latent growth linear mixed effect models. More specifically, we predicted variable-centered emotion pattern scores with time (six administrations of the EV), profile membership and their interaction as fixed factors and included a random intercept for the negative, positive and boredom emotion patterns.^9^ The model for the neutral emotion pattern did not include the interaction term of time and profile membership as the addition of this factor did not improve the model significantly. Results showed significant differences in emotion pattern scores on average for all emotion clusters (all *p* < 0.001). Furthermore, the negative, positive, and boredom pattern scores showed significant linear growth for all participants (all *p* < 0.001). For negative emotion pattern scores (*R*^2^ = 62.99%, fixed effects: *R*^2^ = 40.54%) we found significantly different linear trajectories between the negative profile and the other profiles [compared to neutral profile: β = 0.22, *SE* = 0.05, *t*(877) = 4.57, *p* < 0.001, *VIF* = 4.44; compared to positive profile: β = 0.21, *SE* = 0.05; *t*(877) = 4.16, *p* < 0.001, *VIF* = 4.11; see Figure 4]. Linear growth in positive emotion pattern scores (*R*^2^ = 66.10%, fixed effects: *R*^2^ = 40.44%) were significantly different between the positive and other profiles [compared to neutral profile: β = 0.08, *SE* = 0.04, *t*(877) = 1.99, *p* < 0.05, *VIF* = 3.17; compared to negative profile: β = 0.17, *SE* = 0.05, *t*(877) = 3.43, *p* < 0.001, *VIF* = 2.59]. Boredom pattern scores (*R*^2^ = 56.99%, fixed effects: *R*^2^ = 25.58%) illustrated significantly different linear trajectories between the positive and the neutral profile [β = 0.14, *SE* = 0.05, *t*(877) = 3.14, *p* < 0.010, *VIF* = 3.28].

#### Linking of Co-occurring Emotions and Learning Outcomes

To asses if variable-centered emotion patterns can predict learning gains, separate linear regression models predicting post test score with pretest and variable-centered emotion pattern scores for each point of time were calculated. Results showed that pretest score was a significant predictor of post score in all regressions (β ranging from 0.58 to 0.62; *p* < 0.01). The explanatory value of variable-centered emotion pattern scores beyond the effect of pretest score throughout the different points of time varied. The positive emotions pattern was the only significant predictor besides pretest score for the first administration of the EV [before learning sub goals were set; *F*(5,170) = 26.03, *R*^2^ = 0.42; β = 0.15; *p* < 0.05] and a marginally significant predictor for the second administration [after learning sub goals were set; *F*(5,170) = 25.30, *R*^2^ = 0.41; β = 0.14; *p* = 0.057]. Negative emotions pattern scores significantly predicted post test score for the fourth [second EV during the learning activity; *F*(5,170) = 24.22, *R*^2^ = 0.40; β = −0.13; *p* < 0.05] and sixth administrations of the EV [directly before the post test; *F*(5,170) = 25.08, *R*^2^ = 0.41; β = −0.17; *p* < 0.05] and were a marginally significant predictor for the third [first EV during the actual learning activity; *F*(5,170) = 24.05, *R*^2^ = 0.40; β = −0.11; *p* = 0.086] and fifth EVs [last EV during the learning activity; *F*(5,170) = 23.01, *R*^2^ = 0.39; β = −0.11; *p* = 0.082]. The other patterns showed no significant relation to post test score at any time point.

## Discussion

This study used a person-centered approach to identify emotion profiles and a variable-centered approach to identify variable-centered emotion patterns throughout different phases of a learning session with MetaTutor. We further explored how the emotion profiles and variable-centered patterns identified through these approaches relate to learning outcomes (i.e., through a latent growth linear mixed effect model), and to self-reported habitual emotion regulations strategies.

With the person-centered approach we identified three distinct emotion profiles that reflected different emotional experiences during learning with MetaTutor. In line with our hypotheses and previous research, these profiles included a positive, negative, and neutral (referred to as low intensity in other studies; Robinson et al., 2017) emotion profile. However, it is important to note that the negative profile was not characterized by high levels of negative emotion intensities. It rather represented a group of students that had higher levels of negative emotions than the students belonging to the other profiles. An exception to this pattern was boredom, as the neutral profile showed comparable levels of boredom. This is in line with findings of previous studies emphasizing the distinct role of boredom during learning (Goetz et al., 2014). These findings were further supported through the variable-centered emotion patterns we identified in subsequent steps. Across six points of time throughout the learning session negative and positive emotions remained separate variable-centered patterns from boredom and neutral. This indicates that the separating features of our emotion profiles are related to a stable cluster structure of emotions. Moreover, our results indicated that the most profound difference in emotional experience between emotion profiles were found for the negative emotions (η^2^ = 0.48 for the negative emotion cluster scores as compared to η^2^ = 0.09 for other emotion cluster scores). In our profile solutions negative emotions were associated with one another regardless of their level of arousal. Interestingly, surprise was associated with the negative profile and negative emotions cluster. This finding corresponds with findings of a previous study that found a significant negative relation between surprise and the accuracy of metacognitive judgments indicating a potential negative impact on learning (Taub et al., 2019). However, the lack of differentiation of levels of arousal is likely caused by the imbalanced nature of arousal and valence in emotions measured in the present study (Robinson et al., 2017). Particularly, positive deactivating emotions were underrepresented in the EV. Nonetheless, across two different approaches we identified a theoretically supported and meaningful structure of emotions that centered around three levels of valence—i.e., positive, neutral, and negative.

The most striking feature across all profile solutions was the stability of the negative profile. More specifically, 26 of the 39 (67%) students in the negative profile were always assigned to the same profile regardless of the number of other profiles.^10^ This indicates that the group of students with higher levels of negative emotions is most distinct from all other students (in regard to emotional experience). More importantly, comparisons of the learning outcomes for the profiles revealed that the negative profile performed significantly worse than at least one other profile at post-test in most profile solutions. In the three-profile solution presented in this paper, the negative profile was significantly outperformed by the neutral profile. This finding is well in line with previous studies using person-centered approaches, as multiple studies found that students with negative emotion profiles tend to learn less than those with neutral or positive profiles (Ganotice et al., 2016; Jarrell et al., 2017; Robinson et al., 2017; see Table 2). As opposed to variable-centered approaches that showed positive and negative effects of negative and positive emotions depending on the circumstances, person-centered approaches consistently found detrimental effects of negative emotions for learning. While under certain circumstances single negative (resolved) emotions can potentially benefit learning strategies and outcomes (e.g., D’Mello and Graesser, 2014; Taub et al., 2019), our data provided no support for beneficial effects of experiencing multiple negative emotions (e.g., students that belong to a negative emotion profile). It is important to note that while mixed effects of positive and negative emotions depending on the circumstances have been found in multiple studies, most studies indicate that positive emotions are typically beneficial and negative emotions are detrimental for learning (Boekaerts and Pekrun, 2015). Our results supported this general trend for negative emotions.

In addition to the question of which profiles do significantly differ in learning, we also investigated if and how variable-centered emotion patterns would predict learning. We found that positive emotions before the actual learning activity (EVs 1 and 2, see section Emotion Items) can predict learning outcomes beyond the explanatory effect of prior knowledge. During self-regulated learning with MetaTutor only negative emotions were significant predictors of learning, but not consistently (significant for EV3 and EV6, only marginally significant for EV4 and EV5). These findings indicate that predictive value of variable-centered emotion patterns for learning fluctuates over time and that negative emotions seem to play a predominant role during the learning activity. Furthermore, these finding reflect central approaches related to learning in digital learning environments – products and processes (Garcia-Martin and Garcia-Sanchez, 2018). More specifically, the profile analysis conducted in this study is primarily product focused as we first investigated differences in learning outcomes (i.e., product data) between emotion profiles. With subsequent analyses, we investigated the process nature of emotions by assessing how emotions form patterns over time and how linear developments in these patterns are related to learning.

We faced several challenges and identified limitations when applying the two clustering approaches to the present data. Our sampling approach was defined relative to the start and end of the session. In particular, we selected the first two EVs and the last two in the learning session. Of these questionnaires, only the first in the learning phase (EV3) and the very last before the posttest (EV6) were administered identically for all participants. The EVs in between these were identical relative to the start and end of the learning session, but slightly different in regard to learning time depending on the total number of EVs the participant completed (e.g., for participants with six EVs all questionnaires were in an actual sequence, while for participants with eight EVs the new sequence included the first four EVs and the last two EVs, leaving two EVs out and creating a spline which might not completely reflect the initial temporal trajectory). However, both profile analyses across all time points and the emotion clusters revealed that the selected clusters represented a stable, comparable selection of measures over time.

As a potential explanation for differences between emotion profiles we compared them in regard to emotion regulation and found significant differences in cognitive reappraisal, but not for expressive suppression between profiles. More specifically, the negative profile reported significantly lower habitual use of cognitive reappraisal than the positive profile, but not compared to the neutral profile. To back up these findings we compared the profiles in regard to variable-centered emotion pattern scores and their linear temporal trajectories. We found that emotion profiles did not only differ in averaged emotion pattern scores for all identified emotion patterns but also exhibited significantly different linear growth for negative emotions, positive emotions and boredom (see Figure 4). The most distinct differences lied in the negative emotion pattern as the negative profile displayed a linear increase in negative emotion pattern scores while the scores decreased/stagnated in the other profiles. This illustrates that the negative profile not only starts with higher values of negative emotions, but that this difference got larger over time. Taken together with our finding that the negative emotions cluster negatively predicted learning throughout the learning phase, this indicates that the issues of the negative emotion profile seem to arise over time and are linked to emotion regulation.

A potential explanation for the suboptimal performance of the negative emotion profile is the potential load on working memory imposed by negative emotions and emotion regulation (Curci et al., 2013). While positive emotions cannot enhance working memory beyond its natural capacity, multiple negative emotions may block valuable resources that are particularly required for mastering complex topics and completing challenging learning tasks. This phenomenon might be even more important in digital learning environments as they impose significant challenges to learners (e.g., for navigation through non-linear hyperlinked environments, coordinating multiple goals, integrate agent feedback, use sophisticated learning strategies; Opfermann et al., 2013). Future studies aiming to explain why negative emotions pose a detrimental effect on learning are needed, including cognitive load and its relation to working memory (Seufert, 2018; Anmarkrud et al., 2019).

Another limitation of the present study (and person-centered approaches in general) is the decontextualized nature of emotion measures used. Theories on affective dynamics stretch the importance of specific events or impasses that elicit emotions, however, the events preceding the measurement of emotions have not been considered yet. Specifically, given our data we cannot disentangle whether students learned less because they experienced negative emotions or if they experienced negative emotions because they were having difficulties during the learning process. Identifying if the elevated levels of negative emotions in negative profiles is related to characteristics of the learning task or the learning environment is crucial for both the understanding of the profiles and the development of adaptive systems that can support students and circumvent negative effects of negative emotions on learning though scaffolds. For instance, in our study we cannot rule out that the increase in negative emotion, especially in the negative emotions profile, was related participants being prompted to fill out self-reports to indicate their emotions repeatedly during the learning activity. Likewise, the precedents of emotional reactions during learning should be incorporated in future studies (e.g., by assessing which emotions specific prompts of pedagogical agents elicit). Taub et al. (2019) have shown that facially expressed emotions are associated with the accuracy of learning strategies. Identifying arising negative emotions and the learning processes they directly affect can bridge the gap between emotions and (meta)-cognitive processes. This goes hand in hand with another shortcoming of this line of inquiry – the sole reliance on self-reports to measure emotions. Models and research on emotions clearly state that emotions are multi-faceted processes and limiting our scope to the appraisal component (Scherer and Moors, 2019) is a significant limitation. Building multi-channel, multi-modal emotion profiles through the use of additional data channels can benefit person-centered research by refining profiles and by providing additional explanations how the profiles develop over time (e.g., through peaks in EDA). Lastly, personal predispositions (e.g., personality – narcissism as a predisposition for negative emotionality) is a general cause for differences in emotional experience and emotion regulation, and its effect on learning strategies could be very beneficial to deepen the understanding of emotions in self-regulated learning processes.

## Conclusion

In conclusion, the results of our study highlight the importance of negative emotions during self-regulated learning with digital learning environments during complex learning. The present study adds to research in multiple ways. Methodologically, we have showcased how a person-centered and a novel variable-centered approach complement each other. Particularly identifying variable-centered emotion patterns in addition to emotion profiles enabled us to analyze temporal dynamics of multiple emotions simultaneously. A negative relation between negative emotions and learning outcomes was found with both approaches. This underlines the robustness of this finding and further shows that person-centered and variable-centered approaches can supplement each other. Moreover, clustering approaches offer the possibility to further connect findings from studies using different measures more easily (e.g., achievement emotions vs. learning-centered emotions). Through the combination of person-centered and variable-centered approaches, we have found that both the students with the highest levels of negative emotions overall and higher levels of negative emotions across all students showed a significant negative relation to learning. Furthermore, we have found that these detrimental effects are linked to lower (self-reported) emotion regulation. This indicates the need to identify when elevated levels of negative emotions arise, particularly for students who experience a multitude of negative emotions, for practitioners and researchers to intervene in a timely fashion before the detrimental effects of negative emotions settle in. Specifically, fostering students’ emotion regulation as part of self-regulated learning activities with digital learning environments is a promising prospect to improve students’ emotional experience and learning subsequently. Therefore, the design, development, and implementation of digital learning environments as well as educational interventions should incorporate emotions and emotion regulation as parts of (self-regulated) learning activities to maximize positive effects on students’ learning.

## Data Availability Statement

The datasets generated for this study are available on request to the corresponding author.

## Ethics Statement

This study was approved the by North Carolina State University’s IRB. All subjects gave written consent in accordance with the Declaration of Helsinki.

## Author Contributions

All authors contributed to the conception of the work and revised the final manuscript. RA and MT designed and conducted the study. FW conducted the statistical analyses. FW and MT wrote the first draft of the manuscript. RA and SN provided several rounds of edits on the manuscript.

## Conflict of Interest

The authors declare that the research was conducted in the absence of any commercial or financial relationships that could be construed as a potential conflict of interest.

## Footnotes

1 The dynamics of affective states model and related research often refer to cognitive-affective states instead of emotions. For consistency, readability, and because the cognitive component of these states resembles the appraisal component of emotion theories (e.g., Moors et al., 2013) we will refer to them as emotions. However, we acknowledge arguments that these terms might not be interchangeable in all contexts.

2 Analyses scripts and data are available upon request. For analyses that required (pseudo) randomization, seeds used to obtain the results reported in this paper were documented to guarantee replicability.

3 All subsequent analyses were also conducted for the six- and seven profile-solution. The pattern of results remained similar. These results were not reported in this study due to space constraints.

4 Labels for the profiles were chosen based on the most dominant feature overall and in comparison to the other profiles.

5 The step-wise model selection procedures were not reported due to space constraints. They can be found in the Supplementary Material.

6 This pattern of results was consistent for all profile solutions of the two-step approach but only for the three-profile solution in the initial clustering approach.

7 Further analyses revealed that this pattern of results stayed identical across all profile solutions (four- and five-profile solution from the k-means clustering as well as three- to five-profile solutions from the initial hierarchical clustering analyses).

8 No item had to be rescaled for these analyses, showing that all measures in the pattern correlated positively with the pattern score assigned to each participant.

9 Random slopes were initially considered but lead to potentially overfitted models (singular fit) and were therefore not considered in final analyses. The model selection summary can be found in the Supplementary Material.

10 This pattern was even stronger in the hierarchical cluster analyses as over 90% of students in that profile were consistently assigned to the same profile regardless of the number of other profiles.
