# Supplementary material for: Multiple Negative Emotions During Learning With Digital Learning Environments – Evidence on Their Detrimental Effect on Learning From Two Methodological Approaches
Source: Front Psychol. 2019 Dec 3;10:2678. doi: 10.3389/fpsyg.2019.02678 (PMC6901792; doi:10.3389/fpsyg.2019.02678)
Supplement: Supplementary file 1 [file Table_1.docx]

**Additional information for 5.8.2 Exploring differences in emotion cluster scores between emotion profiles**

**Table S1**

Model description for comparison of emotion clusters scores between emotion profiles

| Model | Description | lmer model syntax |
| --- | --- | --- |
| 0 | Intercept only | [emotion] ~ 1 |
| 1 | Unconditional growth model | [emotion] ~ time |
| 2 | 2 + random intercept | [emotion] ~ time + (1 \|id) |
| 3 | 3 + profile membership | [emotion] ~ time + profile + (1 \|id) |
| 4 | 4 + differing linear changes by profile | [emotion] ~ time + profile + time:profile +  (1 \|id) |

*Note*. [emotion] is a placeholder for scores for the four emotion cluster (negative, positive, boredom, and neutral). Time was coded as the six time points administration of the EV (1-6). Profile: categorical variable indicating profile membership.

**Table S2**

Model comparison for emotion cluster scores

| Comparison | Negative | Positive | Boredom | Neutral |
| --- | --- | --- | --- | --- |
| M0 vs M1 | *Χ*^2^(1) = 0.00;  *p* = 1 | *Χ*^2^(1) = 0.00;  *p* = 1 | *Χ*^2^(1) = 0.00;  *p* = 1 | *Χ*^2^(1) = 0.00;  *p* = 1 |
| M1 vs M2 | *Χ*^2^(1) = 600.77;  *p* < .001 | *Χ*^2^(1) = 681.48;  *p* < .001 | *Χ*^2^(1) = 489.34;  *p* < .001 | *Χ*^2^(1) = 278.72;  *p* < .001 |
| M2 vs M3 | *Χ*^2^(2) = 154.86;  *p* < .001 | *Χ*^2^(2) = 146.59; *p* < .001 | *Χ*^2^(2) = 103.43; *p* < .001 | *Χ*^2^(2) = 20.50; *p* < .001 |
| M3 vs M4 | *Χ*^2^(2) = 23.32;  *p* < .001 | *Χ*^2^(2) = 11.93;  *p* < .010 | *Χ*^2^(2) = 14.26;  *p* < .001 | *Χ*^2^(2) = 3.28;  *p* = .194 |

*Note*. Models for comparison can be found in Table S2.
